# *N*-(2-Methyl­phen­yl)-1,2-benzoselen­azol-3(2*H*)-one

**DOI:** 10.1107/S1600536813024744

**Published:** 2013-09-12

**Authors:** Xu Zhu, Ying Xu, Hongfei Han, Zhiqiang Guo, Xuehong Wei

**Affiliations:** a The School of Chemistry and Chemical Engineering, Shanxi University, Taiyuan 030006, People’s Republic of China; b Department of Chemistry, Taiyuan Normal University, Taiyuan 030031, People’s Republic of China; c Institute of Applied Chemistry, Shanxi University, Taiyuan 030006, People’s Republic of China

## Abstract

In the title Ebselen [systematic name: (2-phenyl-1,2-benzoisoselenazol-3-(2*H*)-one)] analogue, C_14_H_11_NOSe, the benzisoselenazolyl moiety (r.m.s. deviation = 0.0209 Å) is nearly perpendicular to the *N*-arenyl ring, making a dihedral angle of 78.15 (11)°. In the crystal, mol­ecules are linked by C—H⋯O and Se⋯O inter­actions into chains along the *c*-axis direction. The Se⋯O distance [2.733 (3) Å] is longer than that in Ebselen (2.571 (3) Å].

## Related literature

For general background to the properties of Ebselen, see: Bhabak & Mugesh (2010 ▶); Mugesh *et al.* (2001*a* ▶,*b* ▶); Mugesh & Singh (2000 ▶); Engman (1989 ▶); Parnham & Graf (1991 ▶). For related structures, see: Balkrishna *et al.* (2010 ▶); Bhabak & Mugesh (2007 ▶); Chang *et al.* (2003 ▶); Dupont *et al.* (1990 ▶).
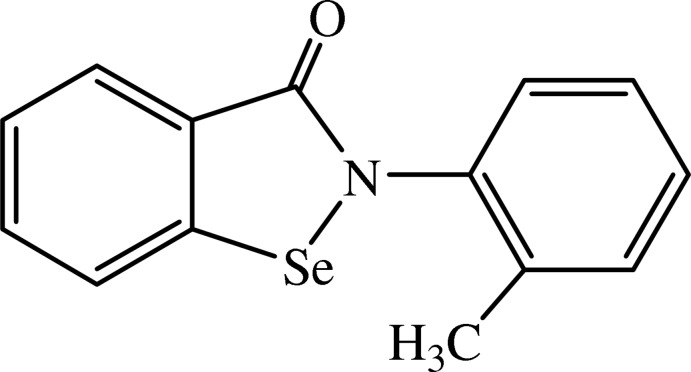


## Experimental

### 

#### Crystal data

C_14_H_11_NOSe
*M* *_r_* = 288.20
Monoclinic, 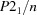

*a* = 7.7319 (14) Å
*b* = 13.491 (2) Å
*c* = 11.913 (2) Å
β = 102.625 (3)°
*V* = 1212.6 (4) Å^3^
*Z* = 4
Mo *K*α radiation
μ = 3.08 mm^−1^
*T* = 273 K
0.30 × 0.20 × 0.20 mm

#### Data collection

Bruker SMART CCD area-detector diffractometer
Absorption correction: multi-scan (*SADABS*; Sheldrick, 1996 ▶) *T*_min_ = 0.459, *T*_max_ = 0.578
4948 measured reflections
2133 independent reflections
1840 reflections with *I* > 2σ(*I*)
*R*_int_ = 0.026

#### Refinement

*R*[*F* ^2^ > 2σ(*F* ^2^)] = 0.041
*wR*(*F* ^2^) = 0.095
*S* = 1.10
2133 reflections
155 parameters
H-atom parameters constrained
Δρ_max_ = 0.52 e Å^−3^
Δρ_min_ = −0.27 e Å^−3^

### 

Data collection: *SMART* (Bruker, 2007 ▶); cell refinement: *SAINT* (Bruker, 2007 ▶); data reduction: *SAINT*; program(s) used to solve structure: *SHELXS97* (Sheldrick, 2008 ▶); program(s) used to refine structure: *SHELXL97* (Sheldrick, 2008 ▶); molecular graphics: *SHELXTL* (Sheldrick, 2008 ▶); software used to prepare material for publication: *SHELXTL*.

## Supplementary Material

Crystal structure: contains datablock(s) I, New_Global_Publ_Block. DOI: 10.1107/S1600536813024744/bg2515sup1.cif

Structure factors: contains datablock(s) I. DOI: 10.1107/S1600536813024744/bg2515Isup2.hkl

Supplementary material file. DOI: 10.1107/S1600536813024744/bg2515Isup3.cml

Additional supplementary materials:  crystallographic information; 3D view; checkCIF report

## Figures and Tables

**Table 1:** Hydrogen-bond geometry (Å, °)

*D*—H⋯*A*	*D*—H	H⋯*A*	*D*⋯*A*	*D*—H⋯*A*
C2—H2⋯O^i^	0.93	2.45	3.132 (4)	130

Symmetry code: (i) 
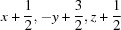
.

**Fig. 1.**
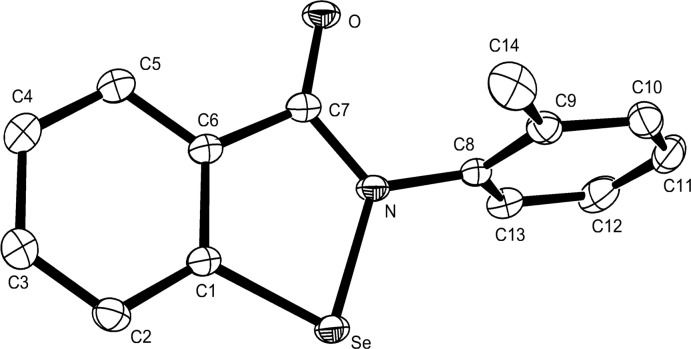
The molecular structure, showing the atom–numbering scheme. Displacement ellipsoids are drawn at the 30% probability level. H atoms have been omitted for clarity.

## supplementary crystallographic information

### 1. Comment

Organoselenium compounds play a very important role in biological and relevant processes. Among them, Ebselen (2-phenyl-1, 2-benzoisoselenazol-3-(2*H*)-one) shows anti-inflammatory, anti-atherosclerotic and cytoprotective properties, and has been used as the most active mimic of GPx (glutathione peroxidase). In the past decade, a number of Ebselen analogues were synthesized and their pharmacological activities were thoroughly studied toward a better understanding of the pharmacology of Ebselen. Furthermore, structurally well defined Ebselen analogues with substituents on *N*-phenyl ring are still rare. Mugesh and co-workers reported several of this kind of Ebselen analogues (Mugesh and Singh, 2000; Mugesh *et al.*, 2001*a*, 2001*b*) which exhibited much higher GPx catalytic activity than that of Ebselen when GSH was used as the co-substrate.

The molecular structure of the title compound is shown in Fig.1. The molecules in the crystal are linked by C-H···O (C2-H2···O[1/2+x,3/2-y,1/2+z]; H···O: 2.45Å; C···O: 3.132 (4)Å, C-H···O: 130°) and Se···O interactions, forming chains along c. The Se···O distance (2.733 (3) Å) is longer than that in Ebselen (2.571 (3) Å, Dupont *et al.*, 1990). The nine-membered benzisoselenazolyl group, which is similar as that of Ebselen, lies on a plane (r.m.s.d. = 0.0209). The dihedral angle between the planes of benzisoselenazolyl group and the N-arenyl ring is 78.15 (11)°, which is much wider than that in Ebselen (33.39 (13)°). The five-membered isoselenazolyl ring is severely strained at the Se atom, the bond length of Se—N [1.876 (3) Å] being shorter than the one in Ebselen [1.896 (3)], while the Se—C1 distance [1.894 (4) Å] and the N—Se—C1 bond angle [85.13 (14) °] are similar to those in Ebselen (1.892 (4)Å and 85.84 (15) °, respectively).

### 2. Experimental

A solution of 2-(chloroseleno)benzoyl chloride (1.27 g, 5 mmol) in dry acetonitrile (25 ml) was added (dropwise and at room temperature ) to a solution of 2-methylaniline (0.536 g, 5 mmol) and triethylamine in dry acetonitrile (15 ml) while stirring during 30 min. The reaction mixture was then further stirred at room temperature for about 5 h and the solvent was evaporated *in vacuo*. The precipitate was filtered off and dried to obtain a yellow solid that was purified in an active neutral alumina column, by using ethyl acetate and chloroform (1:2) as eluent. The resulting yellow compound was recrystallized to obtain yellow blocks for X-ray diffraction analysis. Yield, 79%. mp 175- 176 *^o^*C (173 - 174 ^o^C ^11^). ^1^H NMR (CDCl_3_): δ 2.23 (s, 3 H, CH_3_), 7.28–7.34 (m, 4 H, H—C2, C10, C11, C12), 7.45–7.50 (t, ^3^J = 7.0 Hz, 1 H, H—C4), 7.62–7.72 (m, 2 H, H—C3, C13), 8.12–8.13 (d, ^3^J = 7.8 Hz, 1 H, H—C5); ^13^C NMR: δ 20.9 (CH_3_), 126.7 (C13), 129.1 (C11), 129.6 (C12), 129.8 (C10), 131.8 (C2), 132.1 (C4), 133.9 (C5), 134.4 (C3), 135.1 (C1), 139.4 (C9), 140.3 (C6), 141.6 (C8), 168.4 (C=O); ^77^Se NMR: δ 961. Anal. Calc. For C_14_H_11_NOSe: C, 58.34; H, 3.85; N, 4.86%. Found: C, 58.24; H, 3.81; N, 4.50%.

### 3. Refinement

Crystal data, data collection and structure refinement details are summarized in Table 1. All H atoms were positioned geometrically(C—H = 0.93–0.96 Å), and refined as riding with *U*_iso_(H) = 1.2*U*_eq_ of the adjacent carbon atom (1.5*U*_eq_ for methyl H atoms).

### Crystal data

**Table:**

C_14_H_11_NOSe	*Z* = 4
*M**_r_* = 288.20	*F*(000) = 576
Monoclinic, *P*2_1_/*n*	*D*_x_ = 1.579 Mg m^−^^3^
Hall symbol: -P 2yn	Mo *K*α radiation, λ = 0.71073 Å
*a* = 7.7319 (14) Å	µ = 3.08 mm^−^^1^
*b* = 13.491 (2) Å	*T* = 273 K
*c* = 11.913 (2) Å	Plate, yellow
β = 102.625 (3)°	0.30 × 0.20 × 0.20 mm
*V* = 1212.6 (4) Å^3^	

### Data collection

**Table:**

Bruker SMART CCD area-detector diffractometer	2133 independent reflections
Radiation source: fine-focus sealed tube	1840 reflections with *I* > 2σ(*I*)
Graphite monochromator	*R*_int_ = 0.026
phi and ω scans	θ_max_ = 25.0°, θ_min_ = 2.3°
Absorption correction: multi-scan (*SADABS*; Sheldrick, 1996)	*h* = −7→9
*T*_min_ = 0.459, *T*_max_ = 0.578	*k* = −15→16
4948 measured reflections	*l* = −13→14

### Refinement

**Table:**

Refinement on *F*^2^	Primary atom site location: structure-invariant direct methods
Least-squares matrix: full	Secondary atom site location: difference Fourier map
*R*[*F*^2^ > 2σ(*F*^2^)] = 0.041	Hydrogen site location: inferred from neighbouring sites
*wR*(*F*^2^) = 0.095	H-atom parameters constrained
*S* = 1.10	*w* = 1/[σ^2^(*F*_o_^2^) + (0.0461*P*)^2^ + 0.4966*P*] where *P* = (*F*_o_^2^ + 2*F*_c_^2^)/3
2133 reflections	(Δ/σ)_max_ < 0.001
155 parameters	Δρ_max_ = 0.52 e Å^−^^3^
0 restraints	Δρ_min_ = −0.27 e Å^−^^3^

### Special details

**Table:**

Geometry. All e.s.d.'s (except the e.s.d. in the dihedral angle between two l.s. planes) are estimated using the full covariance matrix. The cell e.s.d.'s are taken into account individually in the estimation of e.s.d.'s in distances, angles and torsion angles; correlations between e.s.d.'s in cell parameters are only used when they are defined by crystal symmetry. An approximate (isotropic) treatment of cell e.s.d.'s is used for estimating e.s.d.'s involving l.s. planes.
Refinement. Refinement of *F*^2^ against ALL reflections. The weighted *R*-factor *wR* and goodness of fit *S* are based on *F*^2^, conventional *R*-factors *R* are based on *F*, with *F* set to zero for negative *F*^2^. The threshold expression of *F*^2^ > σ(*F*^2^) is used only for calculating *R*-factors(gt) *etc*. and is not relevant to the choice of reflections for refinement. *R*-factors based on *F*^2^ are statistically about twice as large as those based on *F*, and *R*- factors based on ALL data will be even larger.

### Fractional atomic coordinates and isotropic or equivalent isotropic displacement parameters (Å^2^)

**Table:**

	*x*	*y*	*z*	*U*_iso_*/*U*_eq_	
Se	1.09550 (5)	0.78392 (3)	0.72071 (3)	0.04308 (16)	
N	0.9365 (4)	0.8320 (2)	0.5900 (2)	0.0409 (7)	
O	0.8278 (4)	0.8041 (2)	0.3987 (2)	0.0515 (7)	
C1	1.1450 (5)	0.6833 (3)	0.6215 (3)	0.0397 (9)	
C2	1.2625 (5)	0.6048 (3)	0.6512 (3)	0.0510 (10)	
H2	1.3234	0.5952	0.7269	0.061*	
C3	1.2857 (6)	0.5417 (3)	0.5648 (4)	0.0621 (12)	
H3	1.3649	0.4892	0.5825	0.074*	
C4	1.1934 (6)	0.5548 (4)	0.4520 (4)	0.0656 (12)	
H4	1.2105	0.5111	0.3951	0.079*	
C5	1.0771 (6)	0.6323 (3)	0.4247 (3)	0.0550 (11)	
H5	1.0157	0.6415	0.3491	0.066*	
C6	1.0513 (5)	0.6967 (3)	0.5096 (3)	0.0407 (9)	
C7	0.9274 (5)	0.7807 (3)	0.4903 (3)	0.0412 (9)	
C8	0.8145 (5)	0.9075 (3)	0.6062 (3)	0.0403 (9)	
C9	0.6444 (5)	0.8811 (3)	0.6142 (3)	0.0489 (10)	
C10	0.5347 (6)	0.9574 (4)	0.6363 (3)	0.0626 (12)	
H10	0.4197	0.9423	0.6423	0.075*	
C11	0.5907 (8)	1.0532 (4)	0.6493 (4)	0.0677 (14)	
H11	0.5142	1.1022	0.6643	0.081*	
C12	0.7594 (8)	1.0778 (3)	0.6403 (4)	0.0682 (13)	
H12	0.7974	1.1433	0.6494	0.082*	
C13	0.8721 (6)	1.0048 (3)	0.6179 (3)	0.0525 (10)	
H13	0.9861	1.0209	0.6107	0.063*	
C14	0.5808 (7)	0.7763 (3)	0.6001 (5)	0.0706 (14)	
H14A	0.5574	0.7586	0.5202	0.106*	
H14B	0.4741	0.7699	0.6282	0.106*	
H14C	0.6701	0.7332	0.6429	0.106*	

### Atomic displacement parameters (Å^2^)

**Table:**

	*U*^11^	*U*^22^	*U*^33^	*U*^12^	*U*^13^	*U*^23^
Se	0.0454 (3)	0.0501 (3)	0.0297 (2)	0.00016 (19)	−0.00047 (16)	−0.00060 (16)
N	0.0423 (17)	0.0472 (17)	0.0286 (16)	0.0005 (15)	−0.0025 (13)	0.0000 (13)
O	0.0548 (17)	0.0605 (17)	0.0323 (14)	0.0018 (14)	−0.0057 (13)	0.0014 (12)
C1	0.039 (2)	0.047 (2)	0.0308 (19)	−0.0045 (17)	0.0041 (16)	−0.0007 (16)
C2	0.052 (2)	0.054 (2)	0.042 (2)	0.005 (2)	−0.0005 (19)	0.0055 (19)
C3	0.063 (3)	0.062 (3)	0.058 (3)	0.014 (2)	0.007 (2)	−0.002 (2)
C4	0.073 (3)	0.070 (3)	0.053 (3)	0.010 (3)	0.011 (2)	−0.015 (2)
C5	0.061 (3)	0.065 (3)	0.037 (2)	0.002 (2)	0.0058 (19)	−0.003 (2)
C6	0.043 (2)	0.046 (2)	0.0316 (19)	−0.0042 (17)	0.0047 (17)	0.0005 (16)
C7	0.040 (2)	0.047 (2)	0.034 (2)	−0.0079 (18)	0.0030 (17)	0.0010 (16)
C8	0.043 (2)	0.047 (2)	0.0281 (18)	0.0034 (18)	0.0023 (16)	0.0016 (16)
C9	0.048 (2)	0.062 (3)	0.036 (2)	0.003 (2)	0.0066 (18)	0.0096 (18)
C10	0.053 (3)	0.094 (4)	0.041 (2)	0.016 (3)	0.012 (2)	0.013 (2)
C11	0.091 (4)	0.070 (3)	0.042 (2)	0.032 (3)	0.015 (2)	−0.001 (2)
C12	0.104 (4)	0.050 (3)	0.047 (3)	0.005 (3)	0.009 (3)	−0.005 (2)
C13	0.059 (3)	0.056 (3)	0.040 (2)	−0.008 (2)	0.004 (2)	−0.0039 (19)
C14	0.061 (3)	0.070 (3)	0.081 (4)	−0.017 (3)	0.016 (3)	0.014 (3)

### Geometric parameters (Å, º)

**Table:**

Se—N	1.876 (3)	C6—C7	1.470 (5)
Se—C1	1.894 (4)	C8—C13	1.384 (5)
N—C7	1.364 (5)	C8—C9	1.386 (5)
N—C8	1.429 (5)	C9—C10	1.394 (6)
O—C7	1.231 (4)	C9—C14	1.494 (6)
C1—C6	1.383 (5)	C10—C11	1.361 (7)
C1—C2	1.389 (5)	C10—H10	0.9300
C2—C3	1.377 (6)	C11—C12	1.373 (7)
C2—H2	0.9300	C11—H11	0.9300
C3—C4	1.388 (6)	C12—C13	1.380 (6)
C3—H3	0.9300	C12—H12	0.9300
C4—C5	1.371 (6)	C13—H13	0.9300
C4—H4	0.9300	C14—H14A	0.9600
C5—C6	1.380 (5)	C14—H14B	0.9600
C5—H5	0.9300	C14—H14C	0.9600
			
N—Se—C1	85.13 (14)	C13—C8—C9	121.7 (4)
C7—N—C8	124.7 (3)	C13—C8—N	118.9 (4)
C7—N—Se	116.6 (2)	C9—C8—N	119.4 (3)
C8—N—Se	117.8 (2)	C8—C9—C10	116.7 (4)
C6—C1—C2	121.4 (4)	C8—C9—C14	121.9 (4)
C6—C1—Se	111.9 (3)	C10—C9—C14	121.3 (4)
C2—C1—Se	126.7 (3)	C11—C10—C9	122.0 (5)
C3—C2—C1	117.7 (4)	C11—C10—H10	119.0
C3—C2—H2	121.1	C9—C10—H10	119.0
C1—C2—H2	121.1	C10—C11—C12	120.4 (5)
C2—C3—C4	121.5 (4)	C10—C11—H11	119.8
C2—C3—H3	119.3	C12—C11—H11	119.8
C4—C3—H3	119.3	C11—C12—C13	119.5 (4)
C5—C4—C3	119.9 (4)	C11—C12—H12	120.2
C5—C4—H4	120.1	C13—C12—H12	120.2
C3—C4—H4	120.1	C12—C13—C8	119.6 (4)
C4—C5—C6	119.9 (4)	C12—C13—H13	120.2
C4—C5—H5	120.1	C8—C13—H13	120.2
C6—C5—H5	120.1	C9—C14—H14A	109.5
C5—C6—C1	119.7 (4)	C9—C14—H14B	109.5
C5—C6—C7	124.3 (3)	H14A—C14—H14B	109.5
C1—C6—C7	116.0 (3)	C9—C14—H14C	109.5
O—C7—N	123.0 (4)	H14A—C14—H14C	109.5
O—C7—C6	126.7 (3)	H14B—C14—H14C	109.5
N—C7—C6	110.3 (3)		

### Hydrogen-bond geometry (Å, º)

**Table:**

*D*—H···*A*	*D*—H	H···*A*	*D*···*A*	*D*—H···*A*
C2—H2···O^i^	0.93	2.45	3.132 (4)	130

Symmetry code: (i) *x*+1/2, −*y*+3/2, *z*+1/2.
